# The Mitochondrial Protein NLRX1 Controls the Balance between Extrinsic and Intrinsic Apoptosis*[Fn FN2]

**DOI:** 10.1074/jbc.M114.550111

**Published:** 2014-05-27

**Authors:** Fraser Soares, Ivan Tattoli, Muhammed A. Rahman, Susan J. Robertson, Antoaneta Belcheva, Daniel Liu, Catherine Streutker, Shawn Winer, Daniel A. Winer, Alberto Martin, Dana J. Philpott, Damien Arnoult, Stephen E. Girardin

**Affiliations:** From the Departments of ‡ Laboratory Medicine and Pathobiology and; § Immunology,; ¶ Saint Michael's Hospital, and; ‖ Department of Pathology, Toronto General Hospital, University of Toronto, Toronto M6G 2T6, Canada and; ** INSERM U1014, Hôpital Paul Brousse, Bâtiment Lavoisier, 14 avenue Paul Vaillant Couturier, 94807 Villejuif Cedex, France

**Keywords:** Apoptosis, Carcinogenesis, Cell Death, Colon Cancer, Innate Immunity, Mitochondria, Nod-like Receptor (NLR), Transformation, NLRs

## Abstract

NLRX1 is a mitochondrial Nod-like receptor (NLR) protein whose function remains enigmatic. Here, we observed that NLRX1 expression was glucose-regulated and blunted by SV40 transformation. In transformed but not primary murine embryonic fibroblasts, NLRX1 expression mediated resistance to an extrinsic apoptotic signal, whereas conferring susceptibility to intrinsic apoptotic signals, such as glycolysis inhibition, increased cytosolic calcium and endoplasmic reticulum stress. In a murine model of colorectal cancer induced by azoxymethane, NLRX1−/− mice developed fewer tumors than wild type mice. In contrast, in a colitis-associated cancer model combining azoxymethane and dextran sulfate sodium, NLRX1−/− mice developed a more severe pathology likely due to the increased sensitivity to dextran sulfate sodium colitis. Together, these results identify NLRX1 as a critical mitochondrial protein implicated in the regulation of apoptosis in cancer cells. The unique capacity of NLRX1 to regulate the cellular sensitivity toward intrinsic *versus* extrinsic apoptotic signals suggests a critical role for this protein in numerous physiological processes and pathological conditions.

## Teaser

**Background:** NLRX1 is a member of the Nod-like receptor family that localizes to the mitochondrial matrix and whose function remains poorly understood.

**Results:** NLRX1 plays a key role in regulating apoptotic cell death and is important in the control of tumorigenesis.

**Conclusion:** NLRX1 regulates the balance between extrinsic and intrinsic apoptosis in cancer cells.

**Significance:** Targeting NLRX1 function could be an interesting strategy against cancer.

## Introduction

The Nod-like receptor (NLR)^3^ family of intracellular sensors plays critical roles in innate immunity in vertebrates (1). One class of NLRs, which includes Nod1, Nod2, and NLRC4, directly detects microbe-associated molecular patterns. Other NLRs, such as NLRP3, detect cellular perturbations that can result from infection or aseptic injury also known as danger-associated molecular patterns. Finally, the NLR proteins NLRC5 and CIITA act as transcriptional regulators for the expression of MHC I and MHC II, respectively.

NLRX1 is a poorly characterized NLR protein that is expressed in mitochondria. Previous reports identified a role for NLRX1 as a negative regulator of antiviral signaling through a direct interaction with the adaptor molecule mitochondrial antiviral signaling protein on the cytosolic side of the mitochondrial outer membrane (2). However, studies from two other groups, including ours, did not confirm these findings (3–5). Instead, we demonstrated that NLRX1 was targeted to the mitochondrial matrix through a functional N-terminal addressing sequence (6), a result that was independently confirmed by other studies (7, 8). In the matrix, we and others observed that NLRX1 interacted with UQCRC2, a matrix-exposed subunit of complex III of the electron transport chain (4, 6). However, because UQCRC2 is also a signal peptidase (9), it is unclear whether this protein is a final binding partner of NLRX1 in the mitochondrial matrix. Finally, in overexpression studies, we demonstrated that NLRX1 expression resulted in the generation of reactive oxygen species (ROS) (10), although it remains unclear whether this effect could have resulted from the jamming of the UQCRC2 signal peptidase or reflected the true function of NLRX1 in mitochondria.

In this study, we identified a critical role for NLRX1 in the control of apoptotic cell death sensitivity in transformed but not primary cells, suggesting a link between NLRX1 and tumorigenesis. In support for this, our *in vivo* studies highlight the importance of NLRX1 in colorectal cancer susceptibility. These observations suggest that targeting NLRX1 might be a valuable approach for cancer therapy.

## EXPERIMENTAL PROCEDURES

### 

#### 

##### Mice

Wild type (WT) and NLRX1 knock-out mice (NLRX1-KO) on a pure C57Bl/6 background were bred in a pathogen-free facility, and animals 6–8 weeks of age were used in this study. Animal studies were conducted under protocols approved by the University of Toronto Committee on Use and Care of Animals. NLRX1-deficient mice have been described previously (5).

##### Azoxymethane (AOM)-induced Colorectal Tumors

NLRX1-deficient and WT mice were injected intraperitoneally once a week for a total of 6 weeks with 10 mg/kg AOM (Sigma). Control animals received saline as the vehicle control. The experiments were performed with at least 8–10 mice in each AOM-treated condition.

##### Induction of Dextran Sulfate Sodium (DSS)-induced Colitis

Colitis was induced with 3% (w/v) DSS (MP Biomedicals) dissolved in sterile, distilled water for experimental days 1–5 followed by normal drinking water until the end of the experiment. Lipocalin-2 was measured by ELISA (R&D Systems) as a marker of inflammation in fecal samples from mice post-DSS treatment. The experiments were performed with at least 8–10 mice in each DSS-treated condition.

##### Colitis-associated Colorectal Cancer

Mice were injected intraperitoneally with 10 mg/kg AOM (Sigma). After 5 days, 3% DSS was given in drinking water over 5 days followed by regular drinking water for 2 weeks. This cycle was repeated twice or three times, and mice were sacrificed 4 weeks after the last DSS cycle. The experiments were performed with at least 8–10 mice in each AOM-treated condition.

##### Cell Culture and Cell Lines

Murine embryonic fibroblast (MEF) cells were cultured in Dulbecco's modified Eagle's medium (Wisent, Canada) supplemented with 10% heat-inactivated fetal bovine serum (FBS; Wisent) and 1% penicillin/streptomycin. Cells were maintained in 95% air and 5% CO_2_ at 37 °C. Generation of primary WT and NLRX1-KO MEFs has been described previously (5). WT and NLRX1-KO MEF transformed cell lines were generated using the SV40 large T antigen. In brief, WT and NLRX1-KO MEFs were transduced with purified SV40 large T antigen Lentifect^TM^ Lentiviral Particles (GeneCopoeia) for 24 h, and puromycin was added to select cells that were positive for the SV40 large T antigen. Cells positive for the SV40 large T antigen were verified by measuring the expression of the SV40 large T antigen by quantitative PCR (qPCR). Similarly, WT and NLRX1-KO SV40-Tschopp MEFs were also monitored for SV40 expression and grown in similar conditions as described above.

##### Cell Death Assays

MEFs were seeded in 96-well plates and stimulated as indicated for 3-(4,5-dimethylthiazol-2-yl)-2,5-diphenyltetrazolium bromide (MTT) assays. Cytotoxicity was measured by adding 20 μl of MTT (12 mm) to cells poststimulation for 60 min. DMSO was added to solubilize the MTT formazan (reduced product) and measured at a wavelength of 570/620 nm. Triplicates were performed for each condition, and results were plotted as means ± S.D. Apoptosis and necrosis were measured using a propidium iodide (PI) and allophycocyanin-conjugated Annexin V Apoptosis Detection kit (eBiosciences). Cells were collected using TrypLE^TM^ (Invitrogen) poststimulation and analyzed by flow cytometry on a BD FACSCalibur according to the manufacturer's protocol.

##### ATP Assay

Intracellular ATP levels were measured by a CellTiter-Glo luminescent cell viability assay (Promega G7571) according to the manufacturer's instructions and read on a Victor3 1420 multilabel automated plate reader (PerkinElmer Life Sciences).

##### ROS Measurements

MEFs were seeded in 6-well plates and stimulated as described. Cells were collected poststimulation with TrypLE (Invitrogen) and stained with 5-(and-6)-chloromethyl-2′,7′-dichlorodihydrofluorescein diacetate (Molecular Probes; 2 μm) to measure reactive oxygen species. Samples were rinsed twice with PBS and analyzed by flow cytometry on a BD FACSCalibur according to the manufacturer's protocol.

##### Luciferase Assays

MEFs were seeded in 96-well plates and stimulated as described. A luciferase-based caspase assay was used to measure caspases-3/7 (Caspase-Glo® 3/7 Assay Systems, Promega) and caspase-8 (Caspase-Glo 8 Assay System, Promega) activity following stimulation according to manufacturer's protocol. Samples were read on a Victor3 1420 multilabel automated plate reader, and triplicates were performed for each condition (means ± S.D. are presented). The luciferase assays in HEK293T cells have been described previously (3), and the human NLRX1 promoter (−1515;+1)-driven luciferase construct was purchased from GeneCopoeia.

##### Histology and Pathological Scoring

Mouse colons were collected, cut open longitudinally, and rolled. Samples were then fixed with 10% (v/v) formalin and stained with hematoxylin and eosin (H&E) at the Toronto Center of Phenogenomics by standard procedures. Pathological scoring was performed blindly by pathologists specialized in intestinal inflammation and carcinogenesis as described previously (11). AOM and AOM/DSS H&E colonic sections were also scored by a pathologist for the degree of dysplasia: aberrant crypt foci, adenomas, high grade dysplasia, and invasive colorectal carcinoma.

##### Real Time qPCR and RT-PCR

Total RNA was extracted using a GeneJET^TM^ RNA Purification kit (Fermentas, Canada) and treated with DNase (Fermentas) to remove traces of genomic DNA. 1 μg of purified RNA was reverse transcribed using Moloney murine leukemia virus reverse transcriptase (Sigma) with random hexamer and oligo(dT) primers (Fermentas). cDNA was diluted accordingly, and 10-μl reactions were set up using Green-2-Go qPCR Mastermix to run qPCR. A CFX384 Touch^TM^ Real-Time PCR Detection System (Bio-Rad) was used to obtain the raw threshold cycle (Ct) values. Results were analyzed using the 2^−ΔCt^ formula normalizing target gene expression to housekeeping control *RPL-19* for mouse samples. Real time qPCR primer sequences are presented in supplemental Table S1. For RT-PCR, 50-μl reactions were prepared using 25 ng of cDNA, and PCR was performed using *Taq* DNA polymerase (Fermentas). PCR products were resolved on a 2% agarose gel. RT-PCR primer sequences are presented in supplemental Table S2.

##### Ectopic Expression in MEFs

The C-terminal FLAG-tagged NLRX1 was developed through PCR using standard cloning techniques. The construct was subcloned into the pHR lentiviral vector through PCR amplification using BamHI and NotI restriction enzymes. NLRX1-FLAG was co-transfected with psPAX2 packaging plasmid and pMD2.G envelope plasmid in 70% confluent HEK293T cells for 48 h to generate lentiviral particles. Wild type and NLRX1-KO SV40 MEFs were subjected to treatments following 4 days of lentiviral transduction. NLRX1 overexpression was detected using an anti-NLRX1 antibody (04-146, Millipore).

##### Histology and Immunohistochemistry

Sections from large intestines were collected from 6-week-old mice. Paraffin sections (5 μm) were deparaffinized in xylene and rehydrated in an ethanol gradient. Antigen retrieval was performed in Rodent Decloaker using a Decloaking Chamber (Biocare Medical, Walnut Creek, CA). Endogenous peroxidase activity was quenched by incubation in 3% H_2_O_2_ for 10 min, and slides were blocked with Dako Protein Block (DakoCytomation Inc., Mississauga, Ontario, Canada) for 30 min at room temperature. The slides were incubated with primary antibody for 1 h at room temperature. The following polyclonal antibodies were used: anti-cleaved caspase-3 (5A1E, 1:800) and Ki-67 (12202, 1:400) (Cell Signaling Technology, Beverly, MA). After washing, slides were incubated with prediluted biotinylated goat anti-rabbit IgGs (Biocare Medical) for 30 min and then treated with 3,3′-diaminobenzidine peroxidase substrate kit (Vector Laboratories, SK-4100). Samples were mounted with aqueous mounting medium (Dako, S3025), and images were taken on a Nikon microscope using NIS-Elements software.

##### Ki-67 and Caspase-3 Quantification

Ki-67- and caspase-3-positive cells in the control or AOM + 3% DSS-treated groups were counted in 100 intact crypts within the colon at a magnification of 400×.

##### Isolation of Mitochondria and in Vitro Assays for the Release of Cytochrome c

Mitochondria were isolated from MEFs by sucrose density gradient centrifugation. Briefly, cells were harvested in phosphate-buffered saline containing 5 mm EDTA, centrifuged at 750 × *g* for 10 min, washed, and resuspended in isotonic mitochondrial isolation buffer (210 mm mannitol, 70 mm sucrose, 1 mm EDTA, and 10 mm HEPES (pH 7.5) supplemented with Complete protease inhibitor mixture). Cells were broken by 10 passages through a 25-gauge needle fitted onto a 5-ml syringe, and the suspension was then centrifuged at 2000 × *g* at 4 °C. This procedure was repeated until nearly all of the cells were broken. Supernatants from each step were pooled before centrifugation (13,000 × *g*, 10 min, 4 °C). The resulting pellet containing mitochondria was resuspended in 1 ml of mitochondrial isolation buffer and layered on top of a discontinuous sucrose gradient consisting of 19 ml of 1.2 m sucrose, 1 mm EDTA, and 0.1% bovine serum albumin in 10 mm HEPES (pH 7.5) over 16 ml of 1.6 m sucrose, 1 mm EDTA, and 0.1% bovine serum albumin in 10 mm HEPES (pH 7.5). Samples were centrifuged (27,000 rpm, 2 h, 4 °C) in a Beckman SW28 rotor. Mitochondria were recovered at the 1.6 m/1.2 m sucrose buffer interface, washed, and resuspended in mitochondrial isolation buffer.

##### Reagents and Antibodies

Sodium azide, 2-deoxy-d-glucose, tunicamycin, thapsigargin, A23187, cycloheximide, rotenone, antimycin A, acetaminophen, AOM, and Z-VAD-fmk were from Sigma-Aldrich. Staurosporine and mouse tumor necrosis factor-α were from Cell Signaling Technology. DSS was from MP Biomedicals. Glucose-free medium was from Wisent. The following antibodies were used to for protein detection: rabbit polyclonal anti-cleaved PARP-1 (Asp-214, Cell Signaling Technology, 9544S), rabbit polyclonal anti-cleaved caspase-3 (Cell Signaling Technology, 9661S), mouse monoclonal anti-tubulin (Sigma, T9026), rabbit polyclonal anti-NLRX1 (Proteintech, 17217-1-AP), and rabbit anti-Hsp60 antibody (Abcam, ab46798).

##### Statistical Analysis

Prism software was used to plot data and determine statistical significance using a Student's *t* test or analysis of variance. Data are presented as means ± S.D. or mean ± S.E. as indicated. A *p* value of <0.05 was considered to be statistically significant.

## RESULTS

### 

#### 

##### NLRX1 Is Glucose-regulated and Down-regulated in SV40-transformed Cells

**FIGURE 1.**
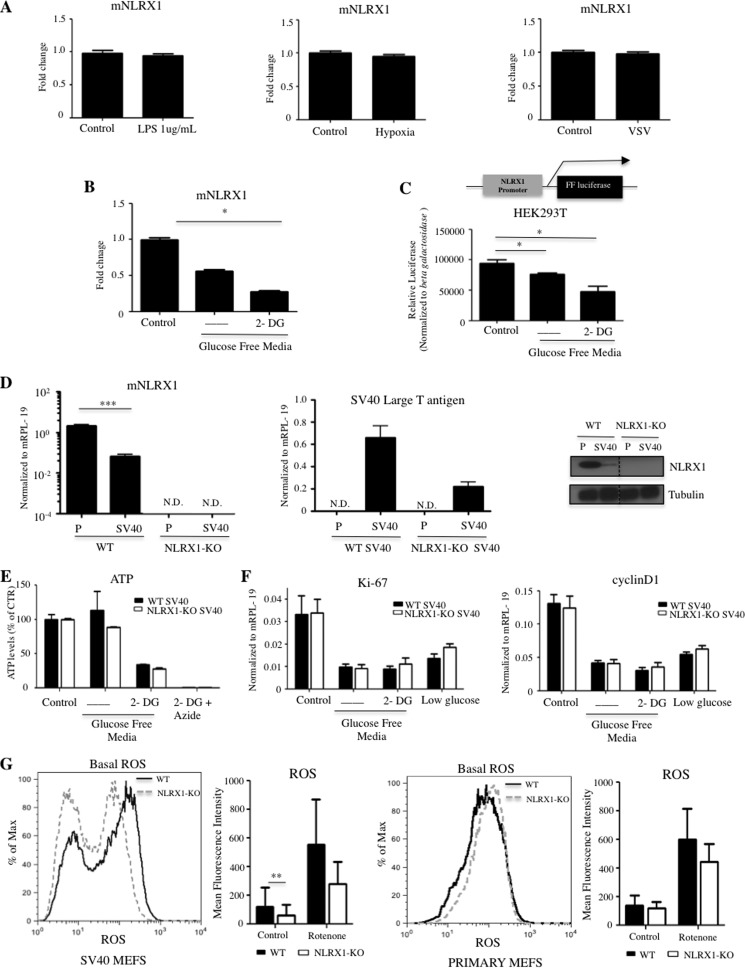
**NLRX1 is a glucose-regulated gene that modulates mitochondrial homeostasis.** *A* and *B*, NLRX1 expression in MEFs treated for 24 h with LPS, hypoxia (1% oxygen), or vesicular stomatitis virus (*VSV*) (*A*) or decreased extracellular glucose alone or in combination with 10 mm 2-deoxyglucose (2-DG) (*B*). *C*, NLRX1 promoter-driven luciferase activity in HEK293T cells stimulated as in *B. D*, NLRX1 and SV40 expression by qPCR and immunoblot in primary (*P*) or SV40-transformed WT and NLRX1-KO MEFs at basal conditions. *E*, ATP levels in SV40-transformed MEFs treated with 10 mm 2-DG alone or in combination with 10 mm sodium azide. *F*, qPCR expression of Ki-67 and Cyclin D1 in resting conditions or following 24-h treatment with 10 mm 2-DG or 1 mm glucose. *G*, measurement of ROS levels in primary or SV40 MEFs at basal conditions or in the presence of rotenone. Data are presented as the mean ± S.D. and are representative of two to four independent experiments (*, *p* < 0.05; **, *p* < 0.01; ***, *p* < 0.0001; *N.D.*, not detectable). In *A* and *B*, *error bars* represent S.E., and in *C–G*, *error bars* represent S.D. *CTR*, control.

To gain insights into the function of NLRX1, we determined its expression by qPCR in MEFs in response to various stimuli. Although NLRX1 expression was unaffected by viral and bacterial infection, lipopolysaccharide treatment, or hypoxia (Fig. 1*A* and data not shown), removal of extracellular glucose or addition of the glycolysis inhibitor 2-deoxyglucose (2-DG) significantly blunted NLRX1 expression (Fig. 1*B*). This effect was likely transcriptional because expression of the luciferase gene under the control of the human NLRX1 promoter was also sensitive to glucose deprivation and 2-DG (Fig. 1*C*). Because cellular transformation is known to profoundly affect glucose metabolism with a reduced role of mitochondria in energy production, we next transformed WT and NLRX1−/− MEFs with the SV40 large T antigen. Strikingly, SV40 transformation resulted in a dramatic reduction of NLRX1 expression at the mRNA and protein levels (Fig. 1*D*), suggesting that NLRX1 activity at the mitochondria might be detrimental to transformed cells. Of note, NLRX1 expression was also found to be profoundly reduced by SV40 or Ras transformation in a genome-wide study (12), thus supporting our results. Although NLRX1 expression was reduced in SV40-transformed cells, we further observed that stimulation with 2-DG also resulted in decreased expression of NLRX1 as was observed in primary MEFs (data not shown). Finally, to demonstrate that low expression of NLRX1 was not the consequence of a glycolysis impairment in our SV40-transformed MEFs, WT SV40-transformed MEFs were stimulated with inhibitors of the mitochondrial respiratory chain (rotenone and antimycin A) to stimulate glycolysis, and we analyzed NLRX1 expression by qPCR. As a control for the efficiency of our stimulations, we also measured the expression of thioredoxin-interacting protein, which is a good marker of glycolysis activity in cells (thioredoxin-interacting protein expression is inhibited by glycolysis and stimulated by glycolysis inhibition). Indeed, thioredoxin-interacting protein expression was strongly blunted by rotenone and antimycin A stimulation, whereas it was strongly induced by 2-DG, thus showing that the mitochondrial inhibitors indeed stimulated glycolysis (data not shown). In those conditions, we did not observe a significant change of NLRX1 expression either in primary or in transformed cells (data not shown).

##### NLRX1 Controls Basal ROS Levels in Transformed MEFs

We measured ATP levels in primary or SV40-transformed cells and found no difference between WT and NLRX1−/− MEFs in resting conditions or in cells treated alone or in combination with 2-DG or sodium azide (NaN_3_), which inhibit glycolysis and mitochondrial respiration, respectively (Fig. 1*E*). In addition, NLRX1-deficient and WT cells displayed similar expression of the proliferation-associated markers Ki-67 and Cyclin D1 in resting conditions or following treatment with low glucose or 2-DG (Fig. 1*F*). Thus, NLRX1 does not appear to directly regulate cellular energy metabolism or proliferation.

We next analyzed mitochondrial parameters in WT and NLRX1−/− SV40-transformed MEFs. The mitochondrial network appeared normal in NLRX1−/− MEFs with no observable fragmentation or collapse (data not shown), suggesting that fission and fusion were unaltered by NLRX1 deficiency in resting conditions. Infection with *Shigella*, which results in a dramatic collapse of the mitochondrial network (13), had similar effects in WT and NLRX1-deficient cells (data not shown). Interestingly, using a dye sensitive to ROS, we observed a significant decrease of basal ROS levels in transformed but not primary NLRX1−/− cells in resting conditions (Fig. 1*G*), although both WT and NLRX1−/− cells mounted a normal ROS response to the mitochondrial inhibitor rotenone (Fig. 1*G*). Thus, NLRX1 is a glucose-regulated gene that controls the tonic levels of ROS in resting conditions, and its expression is strongly blunted by cellular transformation.

##### NLRX1 Protects Transformed but Not Primary Cells against Extrinsic Apoptosis

**FIGURE 2.**
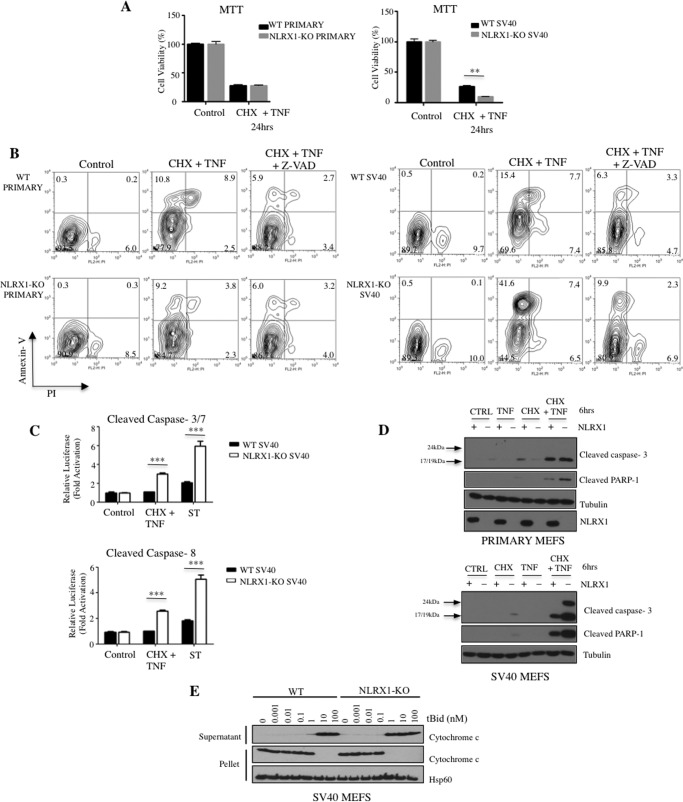
**NLRX1 protects transformed MEFs from extrinsic apoptosis.** *A*, cytotoxicity assays measuring the reduction of the tetrazolium dye MTT in primary or SV40-transformed MEFs treated for 24 h with CHX (10 μg/ml) plus TNF (10 ng/ml). *B*, representative experiments of Annexin V/PI staining of primary and SV40-transformed MEFs treated with CHX (10 μg/ml) plus TNF (10 ng/ml) alone or in combination with Z-VAD-fmk (20 μm). *C*, caspase-3/7 and caspase-8 activation measured in SV40-transformed MEFs treated for 6 h with CHX (10 μg/ml) plus TNF (10 ng/ml) or staurosporine (*ST*; 1 μm). *D*, immunoblot of cleaved caspase-3, cleaved PARP-1, tubulin, and NLRX1 in primary or SV40-transformed MEFs treated for 6 h with CHX ± TNF. *E*, mitochondria isolated from WT or NLRX1-KO MEFs were incubated for 15 min at 30 °C with increasing concentrations (in nm) of recombinant tBid. Mitochondrial and supernatant fractions were then resolved by SDS-PAGE and immunoblotted with an antibody against cytochrome *c*. Equal protein loading of the mitochondrial pellet lanes was monitored using an anti-Hsp60 antibody. Data are presented as the mean ± S.D. and are representative of at least three independent experiments (**, *p* < 0.01; ***, *p* < 0.0001). *Error bars* represent S.D. *CTRL*, control.

Mitochondria play a critical role in apoptosis induced by extrinsic stimuli, such as tumor necrosis factor (TNF). Specifically, extrinsic stimuli activate caspase-8, which results in the insertion of proapoptotic molecules of the Bcl-2 family, such as Bid and Bak, into the outer mitochondrial membrane, causing outer membrane permeabilization, release of cytochrome *c*, and activation of caspase-9 and caspase-3. We stimulated cells with TNF in the presence of cycloheximide (CHX), which is a commonly used trigger for apoptosis mediated by the extrinsic caspase-8-dependent pathway. Interestingly, although primary WT and NLRX1−/− MEFs displayed similar levels of cell death in an MTT assay (Fig. 2*A*), SV40-transformed NLRX1-deficient MEFs exhibited strikingly greater sensitivity to cell death induced by TNF/CHX (Fig. 2*A*). Enhanced cell death in transformed NLRX1−/− cells was due to increased apoptosis as shown in flow cytometry by the fact that Annexin V-positive cells in TNF/CHX-treated NLRX1−/− SV40 MEFs were PI-negative (Fig. 2*B*). Moreover, the pan-caspase inhibitor Z-VAD strongly reduced the Annexin V+/PI− population that accumulated in TNF/CHX-treated NLRX1−/− SV40 MEFs (Fig. 2*B*). To validate these results further, SV40-transformed MEFs were obtained from another group who had independently generated an NLRX1−/− line (4) and are here referred to as SV40 Tschopp-MEFs. Similar increased cell death and apoptosis induction in NLRX1-deficient over WT Tschopp-MEFs were observed following stimulation with TNF/CHX (data not shown). Moreover, a colorimetric assay revealed that caspase-3/7 and caspase-8 induction were more pronounced in SV40-transformed NLRX1−/− MEFs stimulated with TNF/CHX or staurosporine than in WT MEFs (Fig. 2*C*). Western blot analysis using antibodies specific for activated PARP-1 and caspase-3 also supported the increased apoptosis induced by TNF/CHX in SV40-transformed NLRX1−/− MEFs over corresponding WT MEFs, whereas little difference could be observed between primary WT and NLRX1−/− MEFs (Fig. 2*D*). Again, similar results were obtained in SV40-transformed Tschopp-MEFs (data not shown). As a further control that the increased susceptibility to TNF/CHX-induced apoptosis in NLRX1−/− MEFs was due to the lack of NLRX1 expression, WT and NLRX1−/− SV40-transformed MEFs were transduced with lentiviruses expressing NLRX1-FLAG. Ectopic expression of NLRX1-FLAG in NLRX1−/− MEFs resulted in decreased cleavage of PARP-1 and caspase-3 following TNF/CHX stimulation as compared with non-transduced NLRX1−/− MEFs (data not shown).

Finally, we isolated mitochondria from transformed WT and NLRX1−/− MEFs and incubated them with increasing concentrations of purified tBid. Cytochrome *c* release as a consequence of outer mitochondrial membrane permeabilization was analyzed by Western blotting in mitochondrial pellets and supernatants. Interestingly, we observed that cytochrome *c* was released at a lower concentration of tBid in NLRX1−/− mitochondria than in WT mitochondria (Fig. 2*E*), thus showing that the increased sensitivity of NLRX1−/− MEFs to extrinsic apoptosis signals was intrinsic to mitochondria and did not depend on upstream signaling. Of note, the pronounced induction of caspase-8 that we observed in NLRX1−/− MEFS (Fig. 2*C*) likely reflects the fact that autoamplification loops exist during extrinsic apoptosis whereby apoptosis induction results in increased caspase-8 activation (14). Overall, these results demonstrate that NLRX1 protects transformed but not primary MEFs from extrinsic apoptosis signals.

##### NLRX1 Expression Sensitizes Transformed Cells to Intrinsic Apoptosis Signals

**FIGURE 3.**
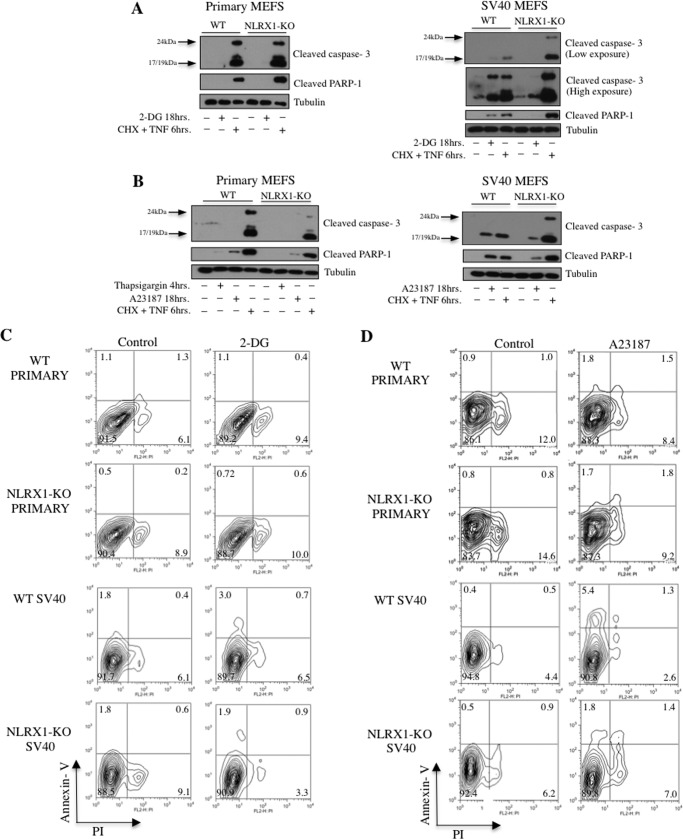
**NLRX1 promotes intrinsic apoptosis.** *A* and *B*, immunoblot of cleaved caspase-3, cleaved PARP-1, and tubulin in primary or SV40-transformed MEFs treated with 2-DG (20 mm) (*A*) or with thapsigargin (3 μm) or the calcium ionophore A23187 (2.5 μm) (*B*) and CHX (10 μg/ml) ± TNF (10 ng/ml). *C* and *D*, Annexin V/PI staining of primary (*C*) and SV40-transformed (*D*) MEFs treated with A23187 (2 μm; 18 h) or 2-DG (20 mm; 18 h).

Sustained inhibition of glycolysis can induce intrinsic apoptosis, in particular in cells that critically rely on glycolysis for the metabolism, such as cancer cells. As for extrinsic apoptosis, intrinsic apoptosis signals converge to the mitochondria, resulting in induction of the cytochrome *c*/caspase-9/caspase-3 cascade. Because NLRX1 expression was down-regulated by glycolysis inhibition, we analyzed its potential role in controlling intrinsic apoptosis. Stimulation of MEFs with 2-DG for 18 h resulted in moderate accumulation of cleaved caspase-3 and PARP-1 in SV40-transformed WT MEFs, whereas primary WT MEFs were resistant to glycolysis inhibition (Fig. 3*A*) in agreement with the fact that transformed cells are commonly more dependent on glycolysis than primary cells. Interestingly, in contrast to our observations with extrinsic apoptosis signals, NLRX1−/− transformed MEFs appeared to be resistant to 2-DG-induced cell death as compared with WT transformed MEFs (Fig. 3*A*).

Increased cytosolic Ca^2+^ concentration can also trigger intrinsic apoptosis. Using the Ca^2+^ ionophore A23187 to increase cytosolic Ca^2+^ concentration, we observed resistance of SV40-transformed NLRX1−/− MEFs and to a lesser degree primary NLRX1−/− MEFs to A23187-induced apoptosis as compared with their WT counterparts as determined by Western blotting against the cleaved forms of PARP-1 and caspase-3 (Fig. 3*B*). For both 2-DG- and A23187-induced apoptosis, these results were confirmed in flow cytometry using Annexin V/PI staining (Fig. 3, *C* and *D*). Finally, transformed and primary MEFs stimulated with 2-DG or A23187 displayed minimal induction of ROS that was similar between WT and NLRX1−/− cells, suggesting that acute ROS induction is not the underlying mechanism of the differential sensitivity of WT and NLRX1−/− MEFs to intrinsic apoptotic stimuli (data not shown).

##### Normal Endoplasmic Reticulum (ER) Stress but Decreased ER Stress-induced Cell Death in NLRX1−/− Cells

**FIGURE 4.**
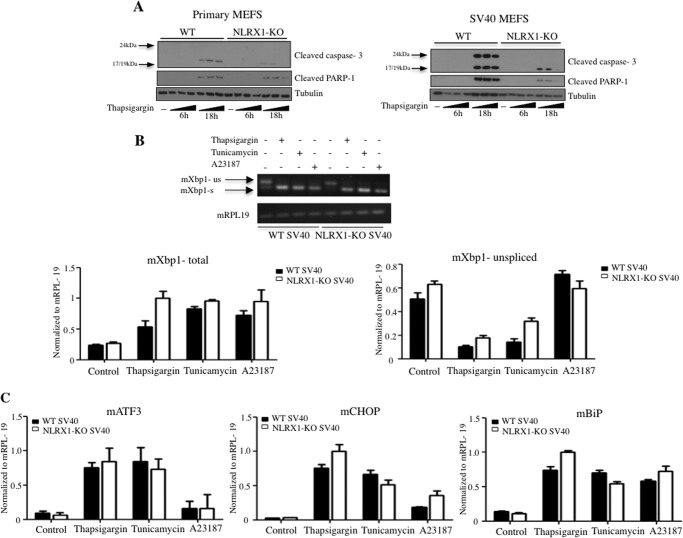
**NLRX1-KO cells exhibit normal ER stress but decreased ER stress-induced cell death.** *A*, immunoblot of cleaved caspase-3, cleaved PARP-1, and tubulin in primary or SV40-transformed MEFs treated for 6 or 18 h with increasing dosages of thapsigargin (1, 5, or 10 μm). *B*, SV40 MEFs were stimulated for 6 h with thapsigargin (1 μm), tunicamycin (1 μg/ml), or A23187 (1 μm), and RT-PCR or qPCR was performed to evaluate the levels of *Xbp1* splicing. *s*, spliced; *us*, unspliced. *C*, mRNA expression of *Atf3*, *Chop*, and *Bip* in MEFs stimulated as described in *B. Error bars* represent S.D.

The above results show that NLRX1−/− MEFs are resistant to apoptotic cell death induced by increased cytosolic Ca^2+^ concentration. A physiological context in which cytosolic Ca^2+^ concentration increases is during ER stress. Using thapsigargin as an inducer of ER stress, we observed that SV40-transformed NLRX1−/− MEFs and to a lesser extent primary NLRX1−/− MEFs were resistant to apoptosis (Fig. 4*A*), a result that mirrors that obtained above with the Ca^2+^ ionophore A23187.

We next wanted to determine whether the resistance of NLRX1−/− MEFs to ER stress-induced apoptosis was caused by a reduced ER stress response. Stimulation of SV40-transformed MEFs with the ER stress inducers thapsigargin and tunicamycin as well as A23187 resulted in increased splicing of *Xbp1* mRNA, a hallmark of ER stress, and similar results were obtained in WT and NLRX1-deficient cells (Fig. 4*B*). Moreover, qPCR analysis of three genes whose expression is regulated by ER stress (*Atf3*, *Chop*, and *Bip*) revealed similar levels of induction in WT and NLRX1−/− MEFs following stimulation with A23187, tunicamycin, and thapsigargin (Fig. 4*C*). Thus, the increased resistance of NLRX1−/− MEFs to ER stress-induced apoptosis is associated with apoptosis regulation rather than the differential induction of ER stress itself.

##### NLRX1 Confers Susceptibility to Colorectal Cancer in the AOM Model

**FIGURE 5.**
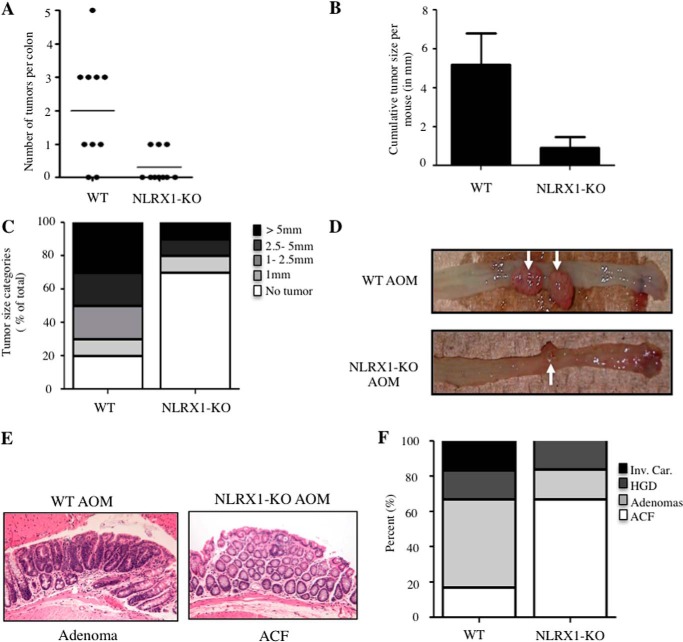
**NLRX1 increases the susceptibility to AOM-induced tumorigenesis.** WT and NLRX1−/− mice were sacrificed 6 months after the last AOM administration. *A–C*, the number of tumors per colon (*A*), cumulative tumor size per mouse (*B*), and percentages of tumor size groups (*C*) were analyzed. *D*, macroscopic observation of tumors (*arrow*) in large bowel from AOM-treated WT (*top*) and NLRX1-KO (*bottom*) mice. *E*, representative H&E micrographs of polyps showing adenoma and aberrant crypt foci (*ACF*) from AOM-treated WT and NLRX1-KO mice, respectively. *F*, histopathological evaluation of aberrant crypt foci (*ACF*), adenomas, high grade dysplasia (*HGD*), and invasive colorectal carcinoma (*Inv. Car.*) that developed in AOM-treated mice. *Error bars* represent S.E.

Our above results suggest that NLRX1 might differentially control apoptotic programs in cancer cells over normal cells, which would affect cancer susceptibility. To test this hypothesis, NLRX1−/− and WT mice received five weekly intraperitoneal injections with the carcinogen AOM, which induces colorectal cancer (CRC), and animals were sacrificed at week 26 after the first AOM injection. Analysis of the intestines of AOM-injected mice revealed a significant reduction in polyp number and size in NLRX1−/− as compared with WT mice (Fig. 5, *A–D*). The tumors were evenly distributed in different cages, and AOM-injected WT and NLRX1−/− mice had comparable microbiota composition (data not shown), thus likely excluding the incidence of microbiota or housing conditions on our results. Histopathology analysis also revealed an overall reduced pathology and absence of high grade carcinoma lesions in NLRX1-deficient mice (Fig. 5, *E* and *F*), suggesting that NLRX1 deficiency reduces cancer progression in this model.

##### NLRX1 Protects against DSS-induced Damage and AOM/DSS-triggered Colorectal Cancer

A variation of the AOM model of CRC has been introduced in which animals receive one injection of AOM followed by several cycles of intestinal injury induced by the chemical irritant DSS (15). Although this AOM/DSS model is now widely used because polyp formation is greatly accelerated, it is unclear whether the two models are equivalent, in particular because DSS induces massive epithelial damage, which exacerbates cell death and repair pathways. Indeed, in this model of CRC, cancer cell expansion likely results from the increased enterocyte proliferation associated with the repair of damaged tissue.

**FIGURE 6.**
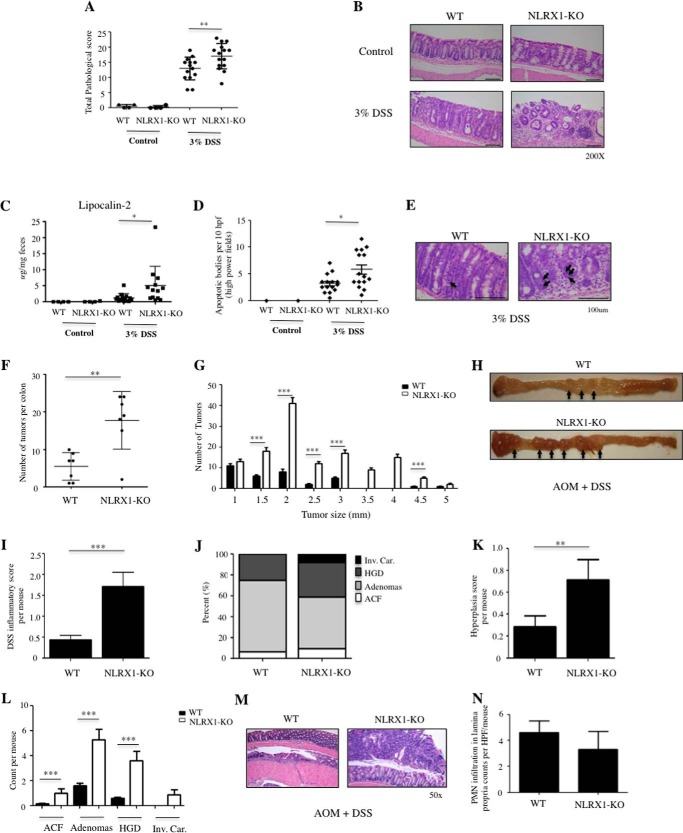
**NLRX1 protects mice against DSS colitis and tumorigenesis induced by AOM and DSS.** *A–E*, WT and NLRX1−/− mice received DSS (3%) in drinking water and were examined at day 7 post-treatment. *A*, histopathological scores. *B*, representative micrographs of H&E-stained distal colonic sections showing increased crypt damage and ulcerations in DSS-treated NLRX1-KO mice (*lower right*) compared with DSS-treated WT mice (*lower left*). *C*, ELISA measurement of lipocalin-2 levels in feces. *D*, quantification of apoptotic bodies observed per 10 high power fields (*hpf*). *E*, representative H&E-stained distal colonic sections from WT and NLRX1-KO mice showing increased epithelial apoptosis in NLRX1-KO mice compared with WT mice. Apoptotic cells display *dark brown*-stained nuclei (*black arrows*). *F–N*, analysis of WT and NLRX1−/− mice in the AOM-DSS model. Total number of tumors (*F*) and tumor sizes (*G*) were analyzed. *H*, representative micrographs showing polyps (*arrow*) in large bowels from AOM-DSS-treated mice. *I–L*, histopathological evaluation displaying scores of inflammation (*I*); percentages of various tumor grades (aberrant crypt foci (*ACF*), adenomas, high grade dysplasia (*HGD*), and invasive colorectal carcinoma (*Inv. Car.*)) (*J*); hyperplasia scores (*K*); and breakdown of the tumors per grade (*L*). *M*, representative micrographs of dysplastic colon sections. *N*, quantitative scoring of neutrophil recruitment. Data are expressed as means ± S.E. (*, *p* < 0.05; **, *p* < 0.01; ***, *p* < 0.001). *Error bars* represent S.E. *PMN*, polymorphonuclear neutrophil; *HPF*, high power field.

When mice were challenged with 3% DSS in drinking water to induce acute colitis, we first observed that NLRX1−/− mice exhibited increased susceptibility as evidenced by histopathological analysis (Fig. 6, *A* and *B*) and quantification of fecal lipocalin-2 (Fig. 6*C*), a marker of colitis (16). Significantly, increased numbers of apoptotic bodies were identified in the intestines of NLRX1−/− over WT mice (Fig. 6, *D* and *E*), suggesting that NLRX1 expression may protect against the cytotoxic effects of DSS.

Next, in a model of AOM/DSS-induced carcinogenesis, we obtained results that were in line with DSS colitis experiments: NLRX1−/− mice displayed increased number of polyps than WT mice (Fig. 6*F*), in particular large tumors (>2 mm) (Fig. 6, *G* and *H*). This phenotype was associated with greater inflammatory (Fig. 6*I*) and pathology scores (Fig. 6, *J–M*) in NLRX1−/− mice, reflecting the increased tissue destruction in these mice, although polymorphonuclear cell infiltration was not different between WT and NLRX1−/− mice (Fig. 6*N*). Thus, these results establish a critical role for NLRX1 in controlling CRC and highlight how differential responses to DSS can affect the outcome of CRC. Similar results were recently reported in the case of MyD88-deficient mice (17).

##### Advanced Apoptosis and Increased Proliferation in Early AOM/DSS in NLRX1−/− Intestinal Crypts

**FIGURE 7.**
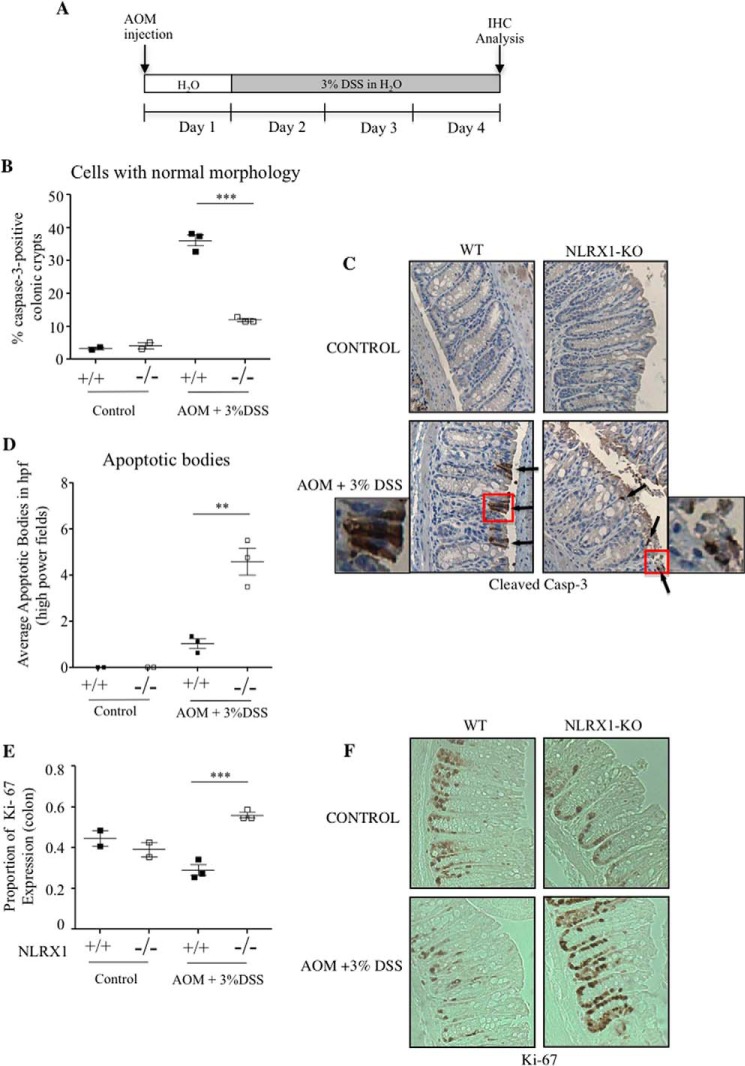
**NLRX1-KO mice exhibit advanced apoptosis and increased proliferation in a short AOM/DSS model.** *A*, schematic illustrating the acute AOM/DSS model. *B*, quantitative analysis of cleaved caspase-3-positive cells by immunohistochemistry (*IHC*). *C*, representative micrographs of colon sections stained for cleaved caspase-3 in WT or NLRX1-KO mice ±AOM/DSS treatment. The *left* and *right panels* are enlargements of caspase-3 (*Casp-3*)-positive cells in WT and NLRX1-KO AOM/DSS-treated mice. *D*, quantitative analysis of apoptotic bodies by capase-3 immunostaining. *E*, quantitative analysis of Ki-67 expression measured with immunohistochemistry. *F*, representative micrographs of colon sections stained for Ki-67 in WT or NLRX1-KO mice ±AOM/DSS treatment. Each data point represents one mouse. Data are expressed as means ± S.E. (**, *p* < 0.01; ***, *p* < 0.001). *Error bars* represent S.E.

To gain insights into the underlying mechanism responsible for increased susceptibility of NLRX1−/− mice to AOM/DSS-induced CRC, we analyzed mice at a very early stage in this model at 3 days into the first round of DSS treatment (Fig. 7*A*). Epithelial erosion was already apparent in NLRX1−/− intestine at this early time point in agreement with the increased susceptibility of NLRX1−/− mice to DSS shown above. Interestingly, WT and NLRX1−/− intestinal crypts displayed strikingly different patterns of cleaved caspase-3 staining in immunohistochemistry. Although caspase-3+ cells in WT mice were elongated and displayed a relatively normal morphological appearance, most caspase-3+ cells in DSS-treated NLRX1−/− mice had the appearance of apoptotic bodies (Fig. 7, *B–D*), suggesting that more NLRX1−/− than WT cells were at an advanced apoptosis stage.

To keep intestinal crypts at a constant length, the rate of enterocyte exfoliation at the luminal side controls stem cell proliferation at the base of the crypts. The analysis of crypt cell proliferation using Ki-67 immunostaining indeed revealed that NLRX1−/− crypts displayed increased proliferation as compared with WT crypts (Fig. 7, *E* and *F*), suggesting that the increased proliferation in NLRX1−/− crypts was likely the consequence of tissue repair induction. The increased susceptibility of NLRX1−/− mice to AOM/DSS colitis is thus likely the consequence of an exacerbated rate of apoptotic cell death in response to DSS treatment followed by increased epithelial proliferation.

## DISCUSSION

In this study, we report the surprising observation that NLRX1 is a glucose-regulated NLR whose expression is also strongly down-regulated in SV40-transformed cells, suggesting that NLRX1 regulates key metabolic processes in the mitochondria. In agreement, we observed that basal levels of ROS were reduced in transformed NLRX1−/− cells. Our observation that NLRX1 expression is regulated by glucose levels but not by bacterial or viral infection is consistent with a metabolism-associated rather than innate immune function of this NLR protein, and this is also supported by the fact that NLRX1 expression was found to be the highest in tissues, such as muscle and heart (10).

Although it remains at this stage unclear how NLRX1 impacts mitochondrial physiology to modulate the balance between intrinsic and extrinsic apoptosis susceptibility, it is possible that it does so by controlling the tonic level of mitochondrial ROS in resting conditions. How NLRX1 regulates basal ROS levels in mitochondria remains to be identified, but the mechanism might be related to the capacity of the molecule to interact with complex III of the electron transport chain, which we and others have reported previously (4, 6). Interestingly, we observed that basal ROS levels were only reduced in transformed but not primary NLRX1−/− MEFs, suggesting a link with the differential apoptosis susceptibility in those cells. Alternatively, it is possible that a difference in basal ROS is not the cause but only a consequence of another, yet unidentified change in mitochondrial physiology that occurs in transformed NLRX1−/− MEFs and that causes differential susceptibility to extrinsic *versus* intrinsic apoptosis signals. For instance, NLRX1 might control the constitutive level of Ca^2+^ uptake to the mitochondria in resting conditions, which could indirectly affect basal ROS levels, and alter apoptosis susceptibility. Importantly, the results that we obtained using isolated mitochondria (see Fig. 2*E*) suggest that, at least in the case of extrinsic apoptotic signals, the NLRX1-dependent control of apoptosis susceptibility is a mitochondrion-intrinsic property and does not depend on upstream signaling. Further studies are required to identify the mechanism by which NLRX1 alters mitochondrial physiology, resulting in regulation of ROS and control of the susceptibility to extrinsic *versus* intrinsic apoptosis signals.

The fact that NLRX1 expression was found to be regulated by glycolysis activity suggests that this protein is likely critical for the fine-tuning of mitochondrial activity in normal cells to adjust metabolic activity to nutrient availability. Nevertheless, our results establish that the lower expression of NLRX1 in transformed MEFs was likely not a direct consequence of glycolysis inhibition. Indeed, given our observations that show lower expression of NLRX1 when glycolysis is inhibited by 2-DG, one would expect that NLRX1 expression should be increased in cancer cells, which rely nearly exclusively on glycolysis for ATP generation, and not decreased if these two observations were directly linked.

At first glance, it seems paradoxical that NLRX1 expression, which we found to be down-regulated by acute glycolysis inhibition induced by 2-DG, would be lower in transformed cells that typically display enhanced glycolysis. We believe that our data, which show that NLRX1 positively regulates apoptosis induced by intrinsic signals, such as glycolysis inhibition, explain this apparent paradox: because cancer cells heavily rely on glycolysis, they are very sensitive to glycolysis inhibition. This is consistent with our data in Fig. 3 that show that 2-DG induces caspase-3 and PARP-1 cleavage in transformed cells but induces weak apoptosis, if any, in primary cells. Thus, lower expression of NLRX1 likely offers a selective advantage to transformed cells to cope with periods of lower supply of glucose and reduced glycolysis. During acute glycolysis inhibition, such as in cells stimulated with 2-DG, the fact that NLRX1 expression drops is likely a cellular adaptation to survive a period of reduced glycolysis.

**FIGURE 8.**
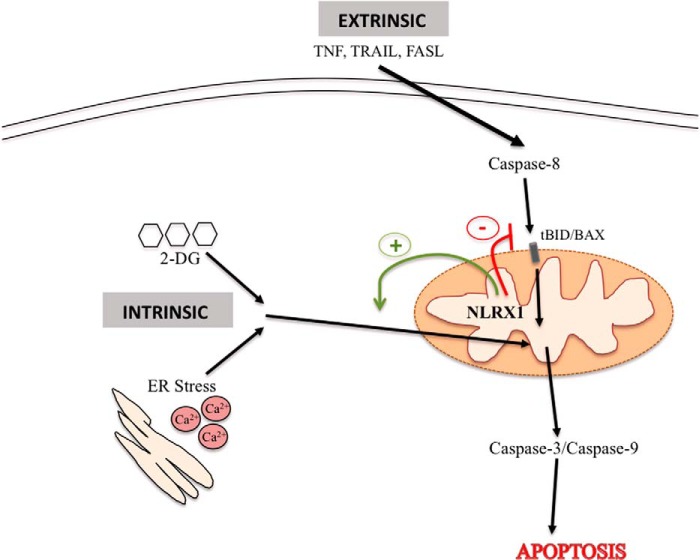
**Model for NLRX1 modulation of apoptosis.** Schematic illustrating NLRX1 regulates the cellular sensitivity toward intrinsic *versus* extrinsic apoptotic signals. *FASL*, FAS ligand.

The point above suggests that NLRX1 expression is likely the result of an equilibrium that is established because of the opposite roles of NLRX1 in the control of the susceptibility to extrinsic *versus* intrinsic apoptosis signals (Fig. 8). This equilibrium would also be affected by the cellular dependence on glycolysis for ATP generation because glycolysis inhibition can trigger intrinsic apoptosis signals if sustained, in particular in glycolytic cells. It will be important to determine whether this hypothesis holds true when comparing the expression of NLRX1 between healthy tissue and tumors *in vivo*. We speculate that NLRX1 expression will reflect the relative exposure to intrinsic apoptosis signals (such as nutrient availability) *versus* extrinsic apoptosis signals (such as inflammation-derived factors, including TNF) in normal tissue or the tumor microenvironment.

There is considerable interest in identifying ways to specifically sensitize tumor cells over normal cells for cancer therapy. For instance, TNF-related apoptosis-inducing ligand (TRAIL), a member of the TNF superfamily, which induces apoptosis in a wide range of cancer cell types, has been considered as an attractive agent for cancer therapy (18). However, many cancers are resistant to TRAIL-based therapies mainly due to the reduced expression of TRAIL receptors and/or the up-regulation of TRAIL pathway-related antiapoptotic proteins. Our results have identified NLRX1 as a mitochondrial protein whose absence specifically sensitizes transformed cells to TNF-induced apoptosis. Understanding the mechanism through which NLRX1 protects transformed cells from apoptosis may thus open up new strategies for cancer therapy. However, these approaches would have to take into consideration the fact that NLRX1 inhibition might also increase the resistance of tumor cells to intrinsic signals, such as nutrient withdrawal.

Although our *in vivo* results clearly identify a critical role for NLRX1 in CRC progression, the fact that we obtained seemingly opposite effects in the two models studied here (AOM and AOM/DSS) complicates the interpretation of the data. However, we believe that these discrepancies are due to the fact that DSS treatment does much more in the AOM/DSS model than simply accelerate tumorigenesis. Unfortunately, very few studies have analyzed the effect of a gene deletion on CRC using these two animal models. Nevertheless, a recent report demonstrated that MyD88-deficient mice, similar to NLRX1−/− mice, exhibited resistance to CRC in an AOM model and increased susceptibility to AOM/DSS-induced carcinogenesis associated with increased apoptosis induced by DSS alone (17). We believe that the apparently contradictory results observed in our study can be explained by the role played by NLRX1 in controlling apoptosis. In the AOM model, we speculate that a reduced rate of intrinsic apoptosis in NLRX1−/− crypts would result in reduced proliferation. Indeed, it is known that the rates of enterocyte apoptosis at the luminal side of the crypt and of stem cell proliferation at the base of the crypt are co-regulated to ensure maintenance of crypt length (19). In turn, reduced proliferation in NLRX1−/− crypts would result in resistance to AOM-induced tumorigenesis. In contrast, in an AOM/DSS model of CRC, inflammation induced by DSS will trigger the accumulation of mediators, such as TNF, which would switch enterocyte apoptosis to an extrinsically driven mechanism and trigger an exacerbated apoptosis in NLRX1−/− crypts. In turn, this would trigger an enhanced proliferation at the base of the crypts to regenerate the damaged tissue, thus likely explaining the increased CRC susceptibility of NLRX1−/− mice in the AOM/DSS model. This model is supported by the data presented in Fig. 7 in which we analyzed crypt proliferation and apoptosis in WT and NLRX1−/− mice in the AOM/DSS model at a very early time point (3 days into the first DSS treatment). Indeed, we observed increased proliferation and a more advanced stage of apoptosis in NLRX1−/− as compared with WT mice in line with the model presented above. Detailed analysis of proliferation *versus* apoptosis in WT and NLRX1−/− intestinal crypts at different stages of tumor development will be critical to provide mechanistic insights into the differential role played by this molecule in AOM and AOM/DSS CRC models.

Our results have shown that NLRX1−/− mice are more susceptible to DSS colitis than WT mice and display enhanced intestinal injury and apoptosis even in the absence of a pretreatment with AOM. This observation suggests that, in contrast to the results obtained in MEFs, NLRX1 deficiency might also affect apoptosis susceptibility in untransformed cells. Because intestinal epithelial cells are among the most highly proliferating cells in the body, it is possible that the results obtained in SV40-transformed *versus* normal MEFs reflect a role for NLRX1 in controlling apoptosis susceptibility in rapidly proliferating cells (or alternatively metabolically active cells) rather than in transformed cells *per se*.

In sum, we have demonstrated that NLRX1 is a mitochondrial protein that controls basal mitochondrial ROS levels as well as the sensitivity to apoptosis in SV40-transformed but not primary MEFs, and the *in vivo* relevance of these findings was evident in murine models of CRC. The fact that NLRX1 deficiency specifically sensitizes transformed cells to apoptotic cell death induced by extrinsic signals, whereas NLRX1 expression sensitizes cells to intrinsic apoptosis stimuli offers promising therapeutic avenues aimed at targeting the unique property of this protein in cancer cells. For instance, inducing cancer cell death by extrinsic apoptotic stimuli (such as TNF or TRAIL) might lead to the selection of cancer cells with increased NLRX1 expression that in turn would be highly susceptible to nutrient withdrawal or intrinsic stresses, such as ER stress. Sequential cycles of extrinsic/intrinsic apoptosis triggers might be a rational strategy against cancer cells.

## Supplementary Material

Supplemental Data

## Footnotes

3 The abbreviations used are:

NLR: Nod-like receptor
MEF: murine embryonic fibroblast
AOM: azoxymethane
DSS: dextran sulfate sodium
ROS: reactive oxygen species
qPCR: quantitative PCR
PI: propidium iodide
Z: benzyloxycarbonyl
fmk: fluoromethyl ketone
2-DG: 2-deoxyglucose
CHX: cycloheximide
ER: endoplasmic reticulum
CRC: colorectal cancer
TRAIL: TNF-related apoptosis-inducing ligand.
