# Supplementary material for: The Mitochondrial Protein NLRX1 Controls the Balance between Extrinsic and Intrinsic Apoptosis
Source: J Biol Chem. 2014 May 27;289(28):19317–30. doi: 10.1074/jbc.M114.550111 (PMC4094044; doi:10.1074/jbc.M114.550111)
Supplement: Supplemental Data [file supp_289_28_19317__index.html]

The Mitochondrial Protein NLRX1 Controls the Balance between Extrinsic and Intrinsic Apoptosis — NLRX1 Controls Apoptotic Cell Death — Supplemental Data 

# The Mitochondrial Protein NLRX1 Controls the Balance between Extrinsic and Intrinsic Apoptosis

## Supplemental Data

**Files in this Data Supplement:**

- Supplemental Tables S1 & S2 - Allowable supplemental Table 1
