# Supplementary material for: The Mitochondrial Protein NLRX1 Controls the Balance between Extrinsic and Intrinsic Apoptosis
Source: J Biol Chem. 2014 May 27;289(28):19317–30. doi: 10.1074/jbc.M114.550111 (PMC4094044; doi:10.1074/jbc.M114.550111)
Supplement: Supplemental Data [file supp_M114.550111_jbc.M114.550111-1.pdf]

The mitochondrial protein NLRX1 controls the balance between extrinsic and intrinsic apoptosis

Fraser Soares[1,#], Ivan Tattoli[1,2,#], Muhammed A. Rahman[1,2], Susan J. Robertson[2], Antoaneta Belcheva[2], Daniel Liu[1], Catherine Streutker[1,3], Shawn Winer[4], Daniel A. Winer[4], Damien Arnoult[5], Alberto Martin[2], Dana J. Philpott[2] and Stephen E. Girardin[1,*]

Supplementary Tables & Figures

Table S1. Real time qPCR primer sequences

Genes	Forward (5'-3')	Reverse (5'-3')
mRpl-19	GCATCCTCATGGAGCACAT	CTGGTCAGC CAGGAGCTT
mNLRX1	TGCCATTTGCCCAGGACCTCTT	GCTCCACTGGATCAAGAAGGAGATATCG
mcyclinD1	GAGATTGTGCCATCCATGC	CTCCTCTTCGCACTTCTGCT
mKi67	GCTGTCCTCAAGACAATCATCA	GGCGTTATCCCAGGAGACT
mChop	CTCCTGTCTGTCTCTCCGGAA	TACCCTCAGTCCCCTCCTCA
mBip	GCCTCATCGGACGCACTT	GGGGCAAATGTCTTGGTT
mAtf3	CGAAGACTGGAGCAAAATGATG	CAGGTTAGCAAAATCCTCAAATAC
SV40	GAAGATGGTGGGGAGAAGAACA	GAGCCTTGGGACTGTGAATCA
mTXNIP	ATCCCAGATACCCCAGAAGC	TGAGAGTCGTCCACATCGTC
mXbp1-total	CCTGAGCCCGGAGGAGAA	CTCG AGCAGTCTGCGCTG
mXbp1-unspliced	CTGACGAGGTTCCAGAGGTG	GCAGAGGTGCACATAGTCTGAG

Table S2. RT- PCR primer sequences

Genes	Forward (5'-3')	Reverse (5'-3')
mXbp1	GGCCTTGTGGTTGAGAACCAGGAG	GAATGCCCAAAAGGATATCAGACTC
mRpl-19	GCATCCTCATGGAGCACAT	CTGGTCAGC CAGGAGCTT
